# Major Determinants of Airway Epithelial Cell Sensitivity to *S. aureus* Alpha-Toxin: Disposal of Toxin Heptamers by Extracellular Vesicle Formation and Lysosomal Degradation

**DOI:** 10.3390/toxins13030173

**Published:** 2021-02-24

**Authors:** Nils Möller, Sabine Ziesemer, Christian Hentschker, Uwe Völker, Jan-Peter Hildebrandt

**Affiliations:** 1 Animal Physiology and Biochemistry, University of Greifswald, Felix Hausdorff-Strasse 1, D-17489 Greifswald, Germany; sabine.ziesemer@uni-greifswald.de (S.Z.); jph@uni-greifswald.de (J.-P.H.); 2 Department of Functional Genomics, Interfaculty Institute for Genetics and Functional Genomics, University Medicine Greifswald, Felix Hausdorff-Strasse 8, D-17475 Greifswald, Germany; hentschkec@uni-greifswald.de (C.H.); voelker@uni-greifswald.de (U.V.)

**Keywords:** *Staphylococcus aureus*, virulence factors, alpha-toxin, transmembrane pores, airway epithelial cells, cellular sensitivity

## Abstract

Alpha-toxin is a major virulence factor of *Staphylococcus aureus*. Monomer binding to host cell membranes results in the formation of heptameric transmembrane pores. Among human model airway epithelial cell lines, A549 cells were most sensitive toward the toxin followed by 16HBE14o^-^ and S9 cells. In this study we investigated the processes of internalization of pore-containing plasma membrane areas as well as potential pathways for heptamer degradation (lysosomal, proteasomal) or disposal (formation of exosomes/micro-vesicles). The abundance of toxin heptamers upon applying an alpha-toxin pulse to the cells declined both in extracts of whole cells and of cellular membranes of S9 cells, but not in those of 16HBE14o^-^ or A549 cells. Comparisons of heptamer degradation rates under inhibition of lysosomal or proteasomal degradation revealed that an important route of heptamer degradation, at least in S9 cells, seems to be the lysosomal pathway, while proteasomal degradation appears to be irrelevant. Exosomes prepared from culture supernatants of toxin-exposed S9 cells contained alpha-toxin as well as low amounts of exosome and micro-vesicle markers. These results indicate that lysosomal degradation of internalized toxin heptamers may be the most important determinant of toxin-resistance of some types of airway epithelial cells.

## 1. Introduction

The apical surface of the human respiratory epithelium is separated from the airspace by the luminal mucus layer [1]. Mucins, salt and water released from secretory cells are major constituents of the airway surface liquid (ASL) which is comprised of two sublayers, a thin (approx. 8 µm) periciliary liquid layer (PCL) of low viscosity and the mucus layer (up to 50 µm) above the PCL that is highly viscous and serves to trap inhaled dust particles and pathogens [2]. Beating of the cilia within the PCL drives the mucus layer including adherent particles towards the pharynx where the entire material is swallowed (mucociliary clearance) [2].

In humans, any inhaled particle is usually disposed of within 2 h so that pathogens are unable to form colonies or biofilms within the airways or to get in touch with the apical cell surfaces of the epithelial cells [3]. In case of disturbances of mucociliary clearance, however, pathogens may remain much longer in the airways, grow and reach higher densities [4,5]. Such a condition may be favorable for commensal and opportunistic bacteria like *S. aureus* which, at higher densities, express soluble virulence factors [6,7]. These molecules are much more mobile in the mucus layer than the bacteria and may readily reach the apical surface of the epithelial cells by diffusion [3].

A major soluble virulence-factor involved in lung pathogenicity of *S. aureus* is alpha-toxin (hemolysin A, Hla; [8]), a pore forming toxin that is secreted by the bacteria as a 33 kDa monomer [9]. The monomers bind to ADAM10, a specific plasma membrane receptor in airway epithelial cells [10,11,12] which facilitates attachment of the toxin to the apical surface of the epithelial cells. Lateral movements of the toxin to plasma membrane areas of high sphingomyelin and phosphatidylcholine contents [13,14] seems to favor multimerization of the toxin molecules and formation of heptameric non-lytic pre-pores [15]. Once the pre-pore is formed, each of the seven monomers rolls out a domain comprised of two beta-sheets that penetrates the plasma membrane. These seven stem domains jointly form a transmembrane beta-barrel channel connecting the host cell cytosol to the extracellular space [15,16].

The alpha-toxin pore is permeable for ions like Na^+^, K^+^ or Ca^2+^ [9,17,18,19] and also for small organic molecules like ATP [20]. In airway epithelial cells, this induces alterations in membrane potential, cytosolic ion concentrations, cell signaling, actin cytoskeleton architecture, in losses of cell-cell and cell-matrix contacts, and, ultimately, in the formation of paracellular gaps in the epithelial cell layer [21,22,23]. In vivo, such effects of pore-formation would disrupt the barrier function of the respiratory epithelium [24].

Epithelial cell types exposed to alpha-toxin display largely different sensitivities toward the toxin. The immortalized human airway epithelial cell line S9 copes well even when in contact with high concentrations of alpha-toxin (2000 ng/mL) while other immortalized cells (16HBE14o^-^) or lung cancer cells (A549) are massively damaged at this concentration [22,25]. In recent studies, it was shown that this can be, at least in part, attributed to the different expression levels of ADAM10 [10,11,12] as well as the differences in sphingomyelin abundances in these cell types [12,14] which makes monomer binding and pre-pore formation more efficient in 16HBE14o^-^ or A549 cells compared with S9 cells [12].

Different sensitivities of cells to pore-forming toxins, however, may also be affected by the different abilities of cells to internalize and degrade or dispose of toxin pores. Endocytosis of pore-containing plasma membrane areas in different types of cultured cells has been previously shown [26]. Especially in human transformed keratinocytes (HaCaT cells), release of pore-containing exosomes has been implicated as a potential pathway for disposal of toxin heptamers while lysosomal degradation was not observed in these cells [26].

The aim of this study was to elucidate whether the observed differences in toxin sensitivities in the airway epithelial cell types may also be attributable to different rates of intracellular processing of pore-containing plasma membrane material, or disposal of such material by release of toxin-containing vesicles to the extracellular space.

## 2. Results

### 2.1. Loss of Heptamers from Airway Epithelial Cells Transiently Exposed to Alpha-Toxin

**Figure 1:**
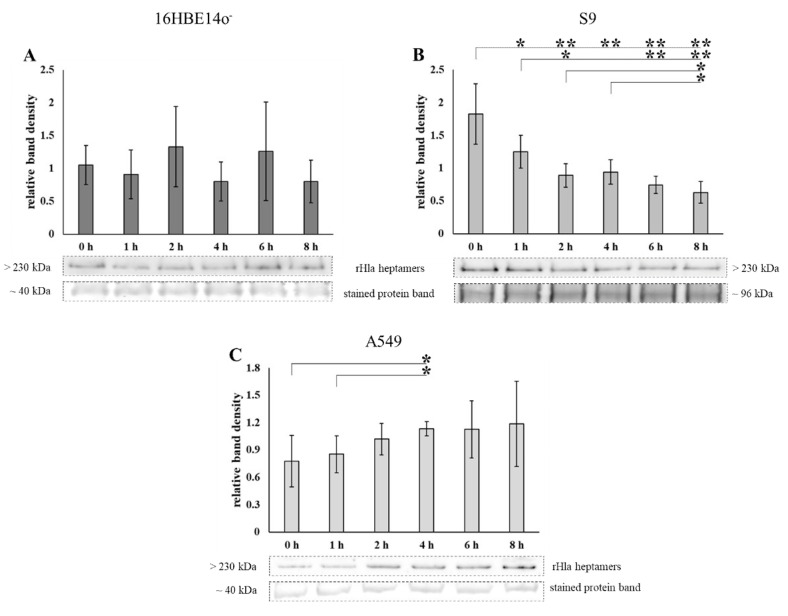
Abundance of residual rHla heptamers in plasma membranes of 16HBE14o^-^, S9, or A549 cells upon rHla-pulse treatment. Due to their different sensitivities against the toxin,16HBE14o^-^ (**A**) were treated with 500 ng/mL rHla, S9 cells (**B**) with 2000 ng/mL rHla and A549 cells (**C**) with 500 ng/mL rHla for 90 min and then incubated for 0, 1, 2, 4 and 8 h in fresh toxin-free cell culture medium. Plasma membrane preparations were generated and the abundances of residual rHla heptamers were analyzed by Western blotting. Mean values of densitometry signals of rHla heptamers were normalized to the densities of the respective Ponceau S stained protein bands (example bands below the graphs). Individual means were tested for significant differences using Student’s *t*-test or Welch’s *t*-test: * = *p* ≤ 0.05, ** = *p* ≤ 0.01 (means ± S.D; *n* = 5).

Pulse-chase experiments were performed by exposing 16HBE14o^-^, S9 or A549 cells, respectively, to 2000 (S9) or 500 ng/mL (16HBE14o^-^ and A549) of recombinant alpha-toxin monomers (rHla) for 90 min and further cultivation of the cells for different periods up to 8 h. The residual amounts of toxin heptamers in plasma membrane preparations of these cells were determined by semi-quantitative Western blotting. The amounts of toxin heptamers remained more or less constant over the 8 h period following toxin exposure in plasma membrane extracts of 16HBE14o^-^ cells (Figure 1A) or even showed a tendency to increase over time in A549 cells (Figure 1C). However, toxin heptamers were lost from cellular membranes in S9 cells following termination of toxin exposure (Figure 1B). These data indicated that the kinetics of toxin heptamer processing were different in these cell types.

### 2.2. Presence of Alpha-Toxin Monomers and Heptamers in Subcellular Fractions of Toxin-Exposed Airway Epithelial Cells

To analyze the distribution of alpha-toxin monomers and heptamers in subcellular membrane fractions of airway epithelial cells upon incubation of cells with recombinant toxin preparations, we treated cells with 2000 ng/mL rHla, lysed them mechanically and fractionated the resulting vesicles according to their densities using ultracentrifugation in continuous 5%–20% iodixanol density gradients [27]. Fraction 1 (F1) contained material of low density (small vesicles), whereas fraction 20 (F20) contained material of high density (large vesicles).

**Figure 2:**
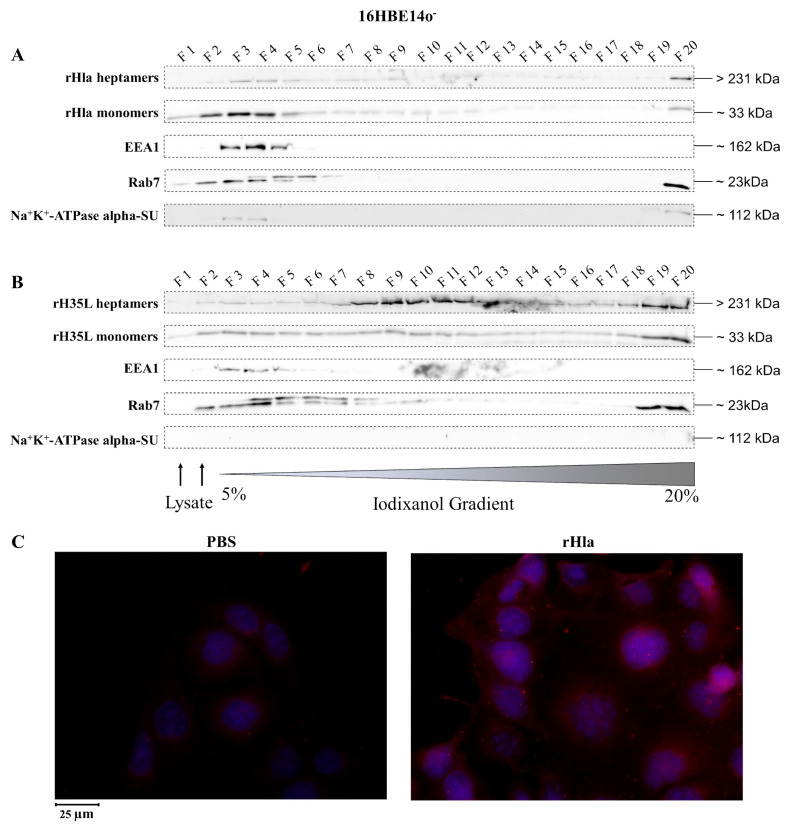
Detection of internalized rHla in 16HBE14o^-^ cells. 16HBE14o^-^ cells were treated for 2.5 h with 2000 ng/mL rHla (**A**) or rH35L (**B**). Cells were mechanically disrupted and post-nuclear supernatants were obtained by centrifugation. These were layered on a continuous iodixanol density gradient (5%–20%). Cellular vesicles and organelles were separated by ultracentrifugation according to their density. Fractions (500 µL) were analyzed by Western blotting for rHla heptamers and monomers, for EEA1 as a marker protein for early endosomes or for Rab7 as a marker protein for late endosomes, autophagosomes or lysosomes. Detection of the alpha-subunit of Na^+^K^+^-ATPase indicated possible contamination of fractions with plasma membrane. Panel (**C**) shows representative examples of fluorescence microscopy images of 16HBE14o^-^ cells incubated with 2000 ng/mL rHla (right) or with PBS as a control (left) for 2 h. Incubation with a Chromeo 546 antibody conjugate against Strep-tag II of rHla was intended to visualize internalized rHla accumulated in vesicles (red staining of vesicles). The cell nuclei were counterstained with 4′,6-Diamidin-2-phenylindol (DAPI). To better see signals in HBE cells, the contrast and brightness of the respective example image of the rHla-treated and PBS-treated cells were adjusted to the same extent.

As shown in extracts of 16HBE14o^-^ cells (Figure 2), alpha-toxin heptamers were present in F2 to F9 as well as in F20 with highest abundances in F3, F4 and F20, while the monomers were more widespread between fraction 1 and 12, as well as in F20 with the highest abundances in F2 to F4 and in F20 (Figure 2A). The marker for early endosomes (EEA1, [28]) was present in F3 to F6. The marker for late endosomes, autophagosomes or lysosomes, Rab7 [29,30] showed a double band around 23 kDa and occurred in F2 to F7, but also in F20. The marker for plasma membrane vesicles, the alpha-subunit of the Na^+^/K^+^-ATPase [31,32] was present in F3 and F4. Some material was also present in F20 (Figure 2A).

The distribution pattern of recombinant alpha-toxin mutant rH35L which binds as a monomer to plasma membranes of eukaryotic cells and forms heptamers like the wild-type toxin, but fails to generate functional transmembrane pores [33], was also analyzed. In extracts of 16HBE14o^-^ cells (Figure 2B), rH35L heptamers were present in virtually all fractions with a focus on F8 to F13, but also on F19 and F20. The rH35L monomers occurred in all fractions with more intense signals in F2 to F5, F8 to F12 and in F19/F20. EEA1, however, showed exactly the same distribution as in extracts of rHla-exposed cells (c.f. Figure 2A). The same was true for Rab7 with the exception that there were faint signals also in F8 to F16. These results indicate that at least fractions of heptameric alpha-toxin had been internalized by 16HBE14o^-^ cells and integrated into intracellular membrane vesicles. Attempts to visualize intracellular rHla by immunofluorescence showed only low signal intensity differences between those cells that were incubated with phosphate-buffered saline (PBS) (controls) compared with those that had been exposed to rHla. However, some intracellular granule-like material could be detected (Figure 2C). This indicated that the amount of internalized material was quite low in 16HBE14o^-^ cells.

**Figure 3:**
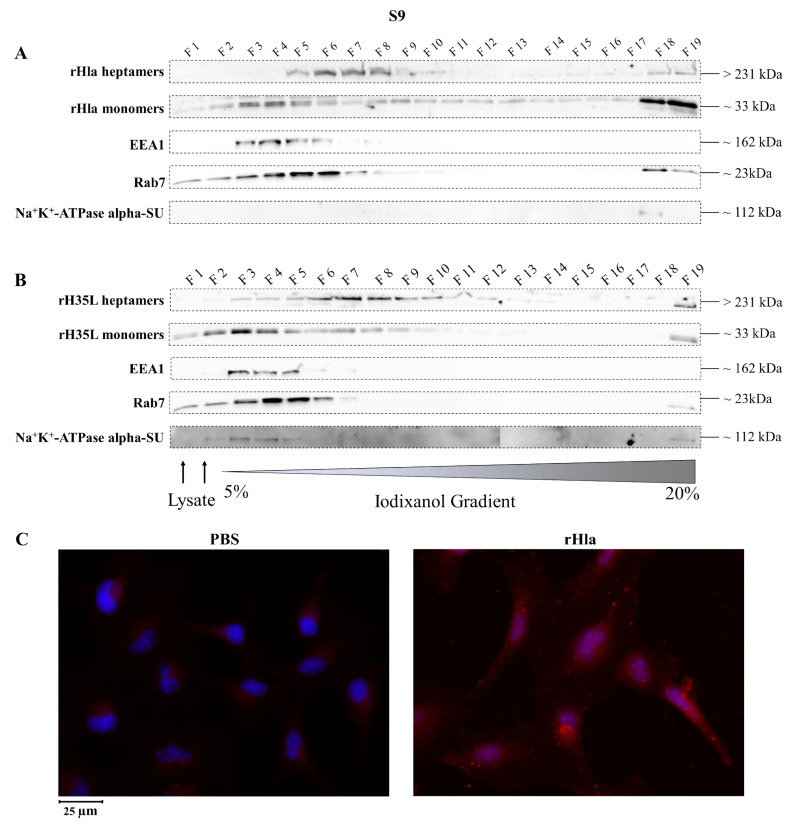
Detection of internalized rHla in S9 cells. S9 cells were treated for 2.5 h with 2000 ng/mL rHla (**A**) or rH35L (**B**). Cells were mechanically disrupted and post-nuclear supernatants were obtained by centrifugation. These were layered on a continuous iodixanol density gradient (5%–20%). Cellular vesicles and organelles were separated by ultracentrifugation according to their density. Fractions (500 µL) were analyzed by Western blotting for rHla heptamers and monomers, for EEA1 as a marker protein for early endosomes or for Rab7 as a marker protein for late endosomes, autophagosomes or lysosomes. Detection of the alpha-subunit of Na^+^K^+^-ATPase indicated possible contamination of fractions with plasma membrane. Panel (**C**) shows representative examples of fluorescence microscopy images of S9 cells incubated with 2000 ng/mL rHla (right) or with PBS as a control (left) for 2 h. Incubation with a Chromeo 546 antibody conjugate against Strep-tag II of rHla was intended to visualize internalized rHla accumulated in vesicles (red staining of vesicles). The cell nuclei were counterstained with DAPI. Note the strong rHla-specific immunofluorescence signals in intracellular granules in alpha-toxin exposed S9 cells (Figure 3C, right panel).

Very similar patterns of monomeric and heptameric alpha-toxin and of markers for subcellular membrane compartments were found in toxin-treated S9 cells (Figure 3). As shown in Figure 3A, alpha-toxin heptamers were present in F5 to F10 as well as in F18/19 with highest abundances in F6 to F8, while the monomers were more widespread with the highest abundances in F3 and F4 as well as in F18 and F19. The marker for early endosomes (EEA1) was present in F3 to F6. The marker for late endosomes or lysosomes, Rab7, showed a double band around 23 kDa and occurred in F3 to F6, but also in F18/19. The alpha-subunit of the Na^+^/K^+^-ATPase was only present in F18.

Heptamers of rH35L were present in virtually all fractions of S9 cell extracts (Figure 3B) with a focus on F6 to F10, but also in F19. rH35L monomers occurred in fractions F1 to F10 with more intense signals in F3 to F5 and in F19. EEA1, however, showed exactly the same distribution as in extracts of rHla-exposed cells (Figure 3A). The same was true for Rab7. The alpha-subunit of Na^+^K^+^-ATPase could be detected in fractions F2 to F5 and F19.

These results indicate that fractions of heptameric alpha-toxin had been internalized by S9 cells and integrated into intracellular membrane vesicles. These results correlated with the occurrence of granulated immunofluorescence signals obtained with antibodies against the recombinant alpha-toxin in toxin-treated S9 cells (Figure 3C).

**Figure 4:**
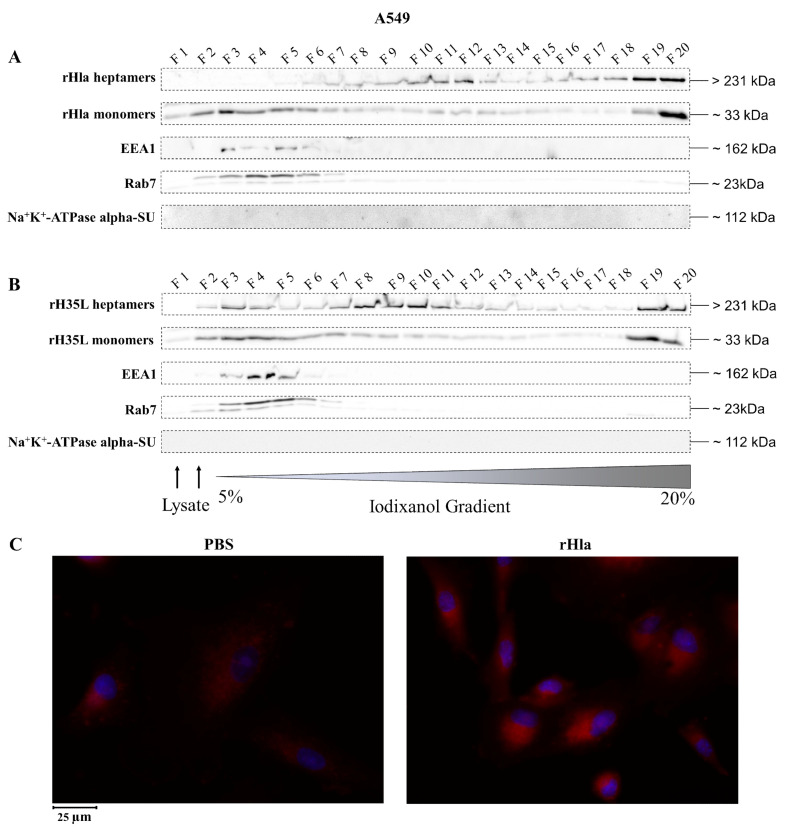
Detection of internalized rHla in A549 cells. A549 cells were treated for 2.5 h with 2000 ng/mL rHla (**A**) or rH35L (**B**). Cells were mechanically disrupted and post-nuclear supernatants were obtained by centrifugation. These were layered on a continuous iodixanol density gradient (5%–20%). Cellular vesicles and organelles were separated by ultracentrifugation according to their density. Fractions (500 µL) were analyzed by Western blotting for rHla heptamers and monomers, for EEA1 as a marker protein for early endosomes or for Rab7 as a marker protein for late endosomes, autophagosomes or lysosomes. Detection of the alpha-subunit of Na^+^K^+^-ATPase indicated possible contamination of fractions with plasma membrane. Panel (**C**) shows representative examples of fluorescence microscopy images of A549 cells incubated with 2000 ng/mL rHla (right) or with PBS as a control (left) for 2 h. Incubation with a Chromeo 546 antibody conjugate against Strep-tag II of rHla was intended to visualize internalized rHla accumulated in vesicles (red staining of vesicles). The cell nuclei were counterstained with DAPI. Note the diffuse rHla-specific immunofluorescence signals in intracellular granules in alpha-toxin exposed A549 cells (Figure 4C, right panel).

Slightly different patterns of heptameric and monomeric alpha-toxin and of markers for subcellular membrane compartments were found in toxin-treated A549 cells (Figure 4). As shown in Figure 4A, alpha-toxin heptamers were present in F7 to F13 as well as in F19 to F20 with highest abundances in F11/12 and F19/20, while the monomers were more widespread with the highest abundances in F2 to F6 as well as in F19/20. The marker for early endosomes (EEA1) was present in F3 to F6. The marker for late endosomes or lysosomes, Rab7, occurred in F2 to F7.

rH35L heptamers in extracts of A549 cells (Figure 4B) were present in virtually all fractions with a focus on F7 to F10, but also in F19/20. The rH35L monomers occurred in all fractions with more intense signals in F2 to F8, but also in F19/20. EEA1 showed exactly the same distribution as in extracts of rHla-exposed cells (Figure 4A). The same was true for Rab7.

These results indicate that at least fractions of heptameric alpha-toxin had been internalized by A549 cells and integrated into intracellular membrane vesicles. Attempts to visualize intracellular rHla by immunofluorescence showed only low signal intensity differences between PBS (controls)-treated and rHla-exposed cells. Cytosolic staining was rather diffuse without any detectable granulation (Figure 4C) indicating that internalization of heptamer-containing plasma membrane material seemed to be sparse in these cells.

### 2.3. Disposal of Alpha-Toxin Heptamers from Airway Epithelial Cells

**Figure 5:**
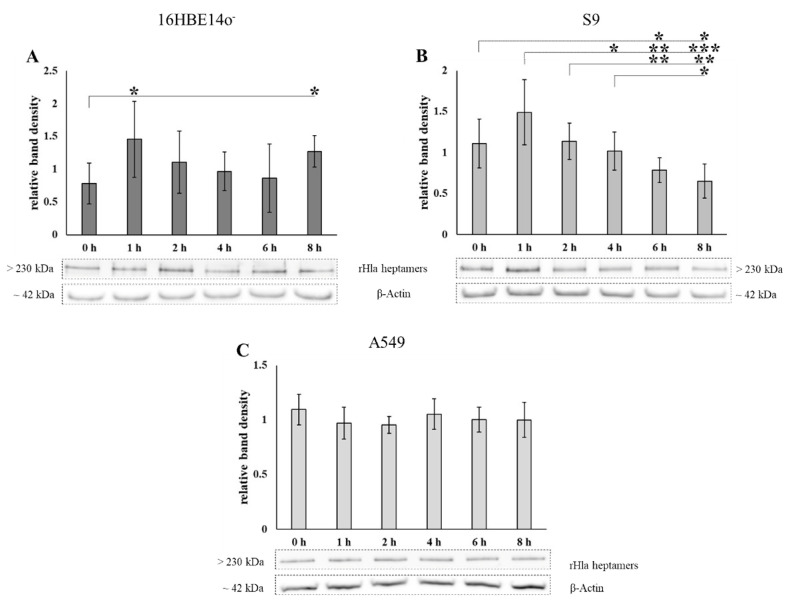
Residual rHla heptamers in whole cell lysates of 16HBE14o^-^, S9- and A549-cells upon pulsed rHla treatment. 16HBE14o^-^ (**A**), S9 (**B**) and A549 cells (**C**) were treated for 90 min with rHla (500 ng/mL for 16HBE14o^-^ and A549 cells; 2000 ng/mL rHla for S9 cells) and then incubated for 0, 1, 2, 4, 8 h in fresh toxin-free cell culture medium. Whole cell lysates were generated and the abundances of residual rHla heptamers were determined by Western blotting. Mean values of densitometry signals of rHla heptamers were normalized to the densities of the respective β-actin protein bands (example bands below the graphs). Individual means were tested for significant differences using Student’s *t*-test or Welch’s *t*-test: * = *p* ≤ 0.05, ** = *p* ≤ 0.01, or *** = *p* ≤ 0.001 (means ± S.D; *n* = 6 for HBE and S9 cells, *n* = 5 for A549 cells).

Pulse-chase experiments using rHla (2000 ng/mL (S9) or 500 ng/mL (16HBE14o^-^ and A549)) were performed as described above, but whole cell extracts were prepared to analyze the total content of alpha-toxin heptamers by semi-quantitative Western blotting. As shown in Figure 5A, the total amount of multimeric Hla in 16HBE14o^-^ cells was more or less constant over 8 h upon termination of toxin exposure. In S9 cells, however, there was a steady decline in heptamer abundance between 1 and 8 h after termination of toxin exposure (Figure 5B). This indicated that S9 cells were able to degrade or expel the toxin. No decline in toxin heptamers was seen in A549 cells (Figure 5C). This indicates that, contrary to S9 cells, 16HBE14o^-^ or A549 cells were not able to efficiently degrade or expel toxin heptamers from the cell.

### 2.4. Degradation of Alpha-Toxin Heptamers through Lysosomal or Proteasomal Pathways?

**Figure 6:**
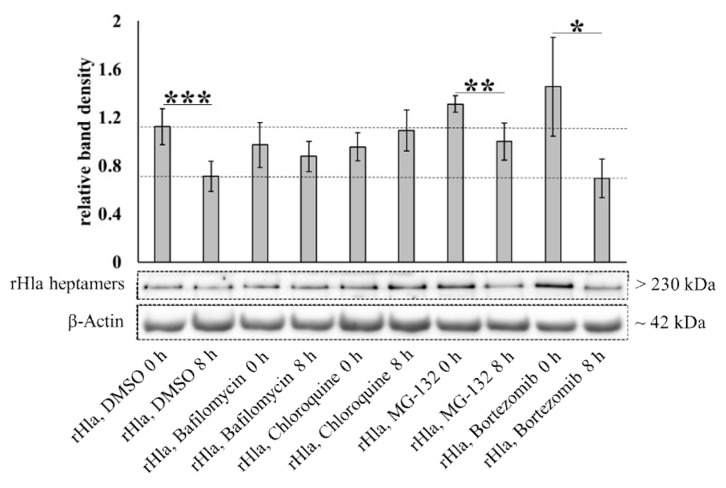
Intracellular degradation of rHla heptamers in alpha-toxin-exposed S9 cells. S9 cells were pre-treated with 100 nmol/L bafilomycin or 100 µmol/L chloroquine to inhibit lysosomal protein degradation or with 10 µmol/L MG-132 or 10 nmol/L bortezomib to inhibit proteasomal protein degradation or with DMSO as a vehicle control for 1 h. Subsequently, cells were exposed to 2000 ng/mL rHla for 90 min. Cells were incubated in fresh toxin-free cell culture medium in the continued presence of the respective inhibitors or DMSO for 0 or 8 h, respectively. Whole cell lysates were generated and used for Western blotting of rHla. Mean values of densitometry signals of rHla heptamers were normalized to the densities of the respective β-actin protein bands (example bands below the graphs). Student’s *t*-test or Welch’s *t*-tests were used to test for significant differences: * = *p* ≤ 0.05, ** = *p* ≤ 0.01, or *** = *p* ≤ 0.001 (means ± S.D; *n* = 5). The broken horizontal lines represent the levels of rHla heptamers in control cells at 0 or 8 h indicating the relative portion of rHla heptamers (approximately 30 % of the original amount) that had been intracellularly digested during this period in S9 cells. Note that bafilomycin- or chloroquine-treatment of cells inhibited heptamer degradation (no significant difference in the heptamer abundances between 0 and 8 h), while treatments of cells with MG-132 or bortezomib did not attenuate the digestion of heptamers during the 8 h incubation period.

S9 cells appeared to be able to remove alpha-toxin heptamers from the cell (Figure 5B). To elucidate whether toxin heptamers were degraded via intracellular proteolysis, we exposed S9 cells to alpha-toxin (2000 ng/mL for 90 min) and cultivated them for another 8 h in the absence or presence of lysosomal inhibitors (100 nmol/L bafilomycin or 100 μmol/L chloroquine) or proteasome inhibitors (10 μmol/L MG-132 or 10 μmol/L bortezomib). The relative amount of toxin heptamers associated with the S9 cells was quantified by semi-quantitative Western blotting on whole cell extracts. Our expectation was that cells should be able to dispose of approximately 45% of the initial amount of toxin heptamers within 8 h (Figure 5B) if none of the potential degradation pathways were inhibited. As shown in Figure 6 this was actually the case (rHla + DMSO, 0 compared with 8 h). In the presence of inhibitors of endosomal or lysosomal functions there was no such decline in toxin heptamer abundance (rHla + Bafilomycin or rHla + Chloroquine, 0 compared with 8 h, each) indicating that these substances suppressed metabolic degradation of the heptamers. However, inhibitors of the proteasomal pathway did not attenuate degradation of toxin heptamers (rHla + MG-132 or rHla + Bortezomib, 0 compared with 8 h, each) indicating that the proteasome does not seem to be a major player in toxin pore removal from the cells.

**Figure 7:**
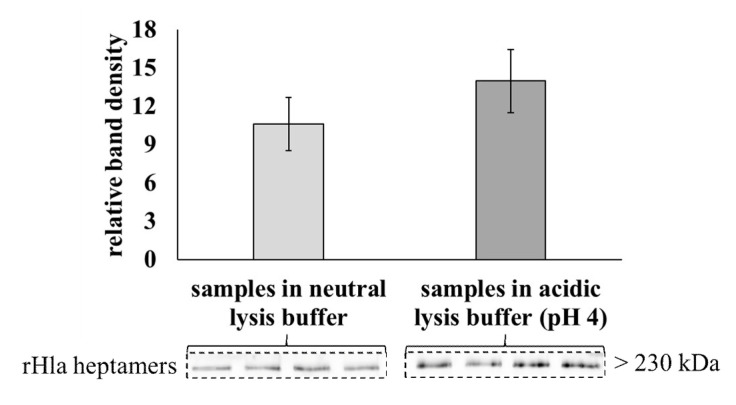
Amounts of residual rHla heptamers in broken cell preparations of 16HBE14o^-^ cells incubated at different pH values of the lysis buffer. 16HBE14o^-^ cells were treated with 2000 ng/mL rHla for 1 h and collected in either a neutral lysis buffer (pH 7.4) or an acidic lysis buffer (pH 4). Whole-cell lysates were generated and used for Western blotting of rHla heptamers. Densitometric signals of rHla heptamers were normalized to the respective sample volume applied to the lane (example bands of each of the 4 replicates below the graphs (means ± S.D.)). Individual means were tested for significant differences using Student’s *t*-test.

To exclude the possibility that the observed loss in toxin heptamers in acidic subcellular compartments did not occur by proteolytic degradation, but just by acid-mediated disassembly of the heptamers, we prepared whole cell lysates of 16HBE14o^-^ cells treated with 2000 ng/mL rHla for 1 h in neutral (pH 7.4) or acidic (pH 4.0) lysis buffers. The relative amounts of toxin heptamers were checked by semi-quantitative Western blotting. As shown in Figure 7 we did not observe any loss in alpha-toxin heptamers in acidic lysis buffer compared with the control assays in neutral lysis buffer. This indicated that toxin heptamers did not disassemble into monomers due to acidic incubation conditions.

### 2.5. Disposal of Alpha-Toxin Heptamers to The Extracellular Space: Exosome/Microsome Formation

An alternative pathway for toxin heptamer removal from cells would be the internalization of pore-containing plasma membrane by endocytosis, formation of multivesicular bodies and exocytosis of exosomes/toxosomes to the extracellular space, as had been previously postulated for alpha-toxin disposal from keratinocytes, liver and kidney cells [26]. Alternatively, budding of toxin-containing plasma membrane areas and formation of micro-vesicles [34] would also remove toxin pores from the cells. To check S9 airway epithelial cells for potential extrusion of toxin-containing vesicular material, we cultured a sub-line of S9 cells under serum-free conditions, incubated these cells with 2000 ng/mL rHla for 90 min, washed the cells thoroughly and cultured them for 8 h in fresh serum-free medium. In a parallel assay, S9 cells were serum-starved in our normal culture medium for 8 or 48 h. As reported previously by other authors [35], such serum-starved cells should develop a stress-response at 8 h and increasing rates of exosome liberation at 48 h. Thus, culture supernatants of such cell preparations were subjected to sequential ultracentrifugation steps to concentrate and finally pellet extracellular vesicular membrane material. Mass spectrometric analysis of supernatants of serum-starved cells should reveal the presence of exosome marker proteins. In supernatants of rHla-exposed cells, however, we expected to find alpha-toxin in addition to the exosomal marker proteins. As reported in Table 1, exosome marker proteins as CD9, CD81, CD151, tetraspanin-6, flotillin-1, Hsp70, Tsg101 and Alix [36,37] were present in the vesicular fraction of supernatants of serum-starved as well as of rHla-exposed S9 cells. In all treatments typical marker proteins for extracellular micro-vesicles were also found (Table 2). In preparations derived from rHla-treated cells, these proteins were detected in combination with rHla (Table S1, full list of proteins). The results indicated that alpha-toxin may indeed be expelled from the cells in the form of exosomes/toxosomes or micro-vesicles by S9 airway epithelial cells. As indicated by the signal log2-ratio of the semi-quantitative comparison of peptide intensities, most of the marker proteins of exosomes (Table S2) and micro-vesicles (Table S3) appeared to be present at higher levels in the pelleted vesicular material of rHla-treated S9 cells than in the samples of 8 h serum-starved cells, but at lower levels than in preparations of cells that had been serum-starved for 48 h.

## 3. Discussion

The differences in relative sensitivities of eukaryotic host cells against pore-forming bacterial toxins [12,22,25,38] may be due to differences in toxin binding to the cell surface, in the rate of heptamerization, in the ability of the cells to internalize membrane areas containing toxin pores, or in the rates of intracellular digestion (lysosomal, proteasomal) or disposal of toxin heptamers to the extracellular space (formation of exosomes/toxosomes or micro-vesicles). The initial interaction of the monomeric toxin with the host cell seems to be facilitated by ADAM10 (a disintegrin and metalloproteinase 10) associated with the outer leaflets of plasma membranes [10,39,40,41]. Thus, the amount of ADAM10 represents a significant factor in the toxin sensitivity of airway epithelial cells [10,11,12]. The assembly of toxin heptamers (non-lytic pre-pores) seems to be critically dependent on the presence of sphingomyelin in the plasma membrane [14], and the abundances of sphingomyelin in the cell membrane of airway epithelial cells seems to have an impact on the sensitivity towards alpha-toxin [12].

Cells, however, may also be able to defend themselves against deleterious actions of pore-forming toxins by removal of pores from the plasma membrane and intracellular degradation or disposal by extruding toxin-containing extracellular vesicles. We reasoned that differences in the ability of different types of airway epithelial model cells to activate such pathways may contribute to the observed differences in toxin sensitivities in such cells.

Exposure of 16HBE14o^-^, S9 or A549 cells to sublethal concentrations of recombinant *S. aureus* alpha-toxin (rHla) for a limited time and analyses of the residual amounts of toxin heptamers in membrane preparations of cells after different cell culture periods (up to 8 h) in the absence of rHla revealed that only S9 cells seemed to be able to remove toxin heptamers from their membranes to a level of approximately one third of the original amount within 8 h (Figure 1B). In 16HBE14o^-^ cells or A549 cells, however, the amounts of toxin heptamers were not significantly altered during the 8 h period following toxin exposure of cells (Figure 1A,C). In A549 cells, the amounts of toxin heptamers even seemed to increase by approximately 35% over the 8 h period following toxin monomer exposure (Figure 1C), indicating that residual monomers that were already attached to the plasma membrane kept forming heptamers and toxin pores after the extracellular rHla-monomers had been removed.

Removal of toxin heptamers from the plasma membrane may occur by endocytosis. To test cells for their abilities to internalize pore-containing plasma membrane areas, we lysed rHla-treated cells and prepared an extract of the intracellular material. Density gradient centrifugation and Western blot analyses of the fractions for the presence of rHla-monomers, toxin heptamers, EEA1 and Rab7 as markers for early or late endosomes, respectively, or Na^+^/K^+^-ATPase alpha subunit as a marker for plasma membrane material revealed that toxin monomers were mainly associated with early endosomes in all three cell types. Toxin heptamers, however, were mostly associated with early endosomes in 16HBE14o^-^ (Figure 2A) or with early and late endosomes in S9 cells (Figure 3A). In A549 cells toxin heptamers were associated with a vesicle fraction of higher density than early or late endosomes (Figure 4A). These results indicate that rHla monomers attached to the extracellular surface of host cells were readily internalized in all three host cell types. However, once toxin heptamers were formed in the plasma membrane, only S9 cells were able to readily channel these membrane areas to the early and late endosomes and, potentially to the lysosomal pathway. To test whether this ability was dependent upon the formation of functional pores or just a consequence of heptamer formation, we treated cells with a Hla-mutant (H35L) which attaches to host cell membranes, builds heptamers, but is unable to form functional pores [33]. Again, the toxin monomers were detected in association with early endosomes, but the heptamers were found in other vesicle fractions of higher densities in all cell types (c.f. Figure 2B, Figure 3B and Figure 4B).

These results indicate that the cellular effects of pore formation [3] may actually have different effects on the internalization and cellular processing of toxin heptamers in the three cell types. The overlap of fractions containing alpha-toxin heptamers and Rab7 in S9 cells (Figure 3A) indicates that endocytosis and intracellular processing functions regularly in these cells. In 16HBE14o^-^ or in A549 cells, however, there is no overlap of alpha-toxin and Rab7 containing vesicles (Figure 2A and Figure 4A) which indicates that toxin heptamers are either not internalized at all or that internalization and processing occurs via other pathways than the regular endosomal/lysosomal pathway. This conclusion is supported by the results obtained with rHla-treated cells, in which rHla was detected by immunofluorescence. Only in S9 cells, were rHla-specific signals clearly associated with intracellular vesicles (Figure 3C), while only low numbers of granulated signals were detectable in 16HBE14o^-^ cells (Figure 2C). In A549 cells, signals were diffusely distributed across the cell with no granule-like structuring (Figure 4C).

Cells may process internalized membrane material in different ways, either by intracellular degradation of internalized proteins or by formation of multivesicular bodies and exocytosis of exosomes or budding of micro-vesicles, which allows disposal of unwanted membrane proteins to the extracellular space. Intracellular degradation of internalized proteins may occur through the lysosomal pathway or by proteasomal digestion. To identify the pathway used by S9 airway epithelial cells to degrade internalized toxin heptamers, we exposed cells to rHla and cultured them for another 8 h in the absence of rHla, but in the presence of inhibitors of the lysosomal pathway (bafilomycin, chloroquine) or the proteasome (MG-132, bortezomib). As shown in Figure 6, MG-132 or bortezomib did not suppress the loss of toxin heptamers over the 8 h period after rHla-exposure of cells that was observed in control cells without inhibitors. This indicates that the proteasome is most likely not involved in the intracellular degradation of internalized toxin heptamers in S9 cells.

Bafilomycin inhibits the proton-pumping V-ATPase in the membrane of intracellular lysosomes and lysosomal acidification [42]. chloroquine may function as a proton buffer within lysosomes [43], but may also have other mechanisms of action to inhibit lysosomal function [44]. Both substances inhibited the degradation of rHla heptamers in S9 cells (Figure 6). This indicates that the main route of toxin heptamer degradation of internalized alpha-toxin pores in S9 cells is via the lysosomal pathway. To make sure that toxin heptamer removal from the cells actually occurs via protein digestion of heptamers and not just by acid-mediated disintegration of heptamers into monomers and digestion of the monomers, we incubated cellular extracts containing rHla heptamers at either neutral (pH 7) or acidic (pH 4, lysosomal pH). As shown in Figure 7, we did not observe any differences in the abundances of rHla-heptamers after the incubation, which indicates that rHla-heptamers are stable irrespectively of the environmental pH in the range between 4 and 7. This means that S9 cells are able to internalize membrane areas containing toxin heptamers and degrade these heptamers using the lysosomal degradation machinery.

Finally, we wanted to test whether S9 cells are able to dispose of rHla-heptamers into the extracellular space by generating pore-containing extracellular vesicles (exosomes/toxosomes or micro-vesicles). To this end, cells were exposed for 90 min to rHla, washed and cultured for another 8 h in fresh culture medium. Exosomes and micro-vesicles in the supernatant of such cell cultures were prepared by ultra-centrifugation and protein content was analyzed by LC-MS. Supernatants of S9 cells that had been serum-starved for 8 or 48 h, respectively, were used to compare the protein patterns with that in supernatants of rHla-treated cells, as serum-starvation had been found to induce exosome generation [35]. Proteomic analyses showed that supernatants of rHla-treated and serum-starved cells showed different patterns of proteins (Table S1), but were very similar in those protein groups that have been discussed [36,37] as markers for exosomes (Table 1), or micro-vesicles (Table 2). In supernatants of rHla-treated cells, Hla-specific mass spectrometric signals were clearly identified is association with the proteins listed in the Table 1 and Table 2 and showed a higher amount of most of these marker proteins than the 8 h starving cells (Tables S2 and S3). This indicates that exposure of cells to *S. aureus* alpha-toxin or serum-starvation are stressful conditions to S9 cells which induce the formation of extracellular vesicles, and that relocation of membrane material containing alpha-toxin pores to the extracellular space via exosome/micro-vesicle formation seems to contribute to removal of rHla-heptamers from S9 cells. Which of the two pathways for heptamer disposal in S9 cells may be more efficient is still an open question and will be addressed in the future.

The differences in the abilities to use this pathway for toxin removal from the cells seem to contribute to the different sensitivities of airway epithelial model cell types toward *S. aureus* alpha-toxin.

## 4. Materials and Methods

### 4.1. Chemicals and Reagents

Chloroquine diphosphate salt (C6628), DMSO (dimethyl sulfoxide, D2650), KH_2_PO_4_ (P0662), sodium deoxycholate (D6750), NaH_2_PO_4_ (S0751), sodium orthovanadate (S6508) and saponin (S2149) were obtained from Sigma Aldrich (Steinheim, Germany). bafilomycin A1 (10-2060) was ordered from Focus Biomolecules (Plymouth Meeting, PA, USA), bortezomib (2204S) from Cell Signaling (Frankfurt am Main, Germany) and Mg-132 (S2619) from Selleckchem.com (Munich, Germany). KOH (105033) was purchased from Merck (Darmstadt, Germany) and trypsin (including ethylene diamine tetra-acetic acid, EDTA) (L2143) from Biochrom (Berlin, Germany), OptiPrep™ (60% iodixanol [*w*/*v*], 07820) from STEMCELL Technologies (Köln, Germany) and pep-statin A (A2205) from PanReacAppli-Chem (Darmstadt, Germany). WesternBright chemiluminescence substrate from Advansta (K-12045-D50) was ordered from Biozym (Oldendorf, Germany).

Antibodies (Ab) were obtained from these sources: Hla-Ab (S7531) from Sigma (Steinheim, Germany); EEA1-Ab (sc-137130) and mouse IgGκ light chain binding protein (sc-516102) from Santa Cruz Biotechnology (Heidelberg, Germany); Rab7-Ab (PA5-52369) from Thermo Fisher Scientific (Bonn, Germany); Na^+^K^+^-ATPase α SU-Ab (AB_2166869) from Developmental Studies Hybridoma Bank (Iowa City, IA, USA); StrepMAB-Classic Chromeo 546-Konjugat (2-1550-050) from iba (Göttingen, Germany); goat anti-rabbit IgG-HRP (7074s) and horse anti-mouse IgG-HRP (7076S) from Cell Signaling (Frankfurt am Main, Germany). All other chemicals were reagent grade and obtained from Roth (Karlsruhe, Germany).

### 4.2. Expression and Purification of Recombinant S. aureus Alpha-Toxin (rHla) and rH35L

Recombinant alpha-toxin (rHla) was prepared and purified as described previously [45]. Purity of the toxins was verified by SDS-PAGE and Coomassie brilliant blue staining. Biological activities of rHla were tested in a hemolysis assay in sheep blood agar. rH35L is a recombinant Hla in which the amino acid histidine at the 35th position has been replaced by a leucine, whereby rH35L can still form heptamers, but is not able to form a transmembrane pore in the plasma membrane of cells [33]. The plasmid for the preparation of rH35L was designed by Dr. Christian Müller (University of Greifswald, Germany) [19] and preparation and purification of rH35Lwas equivalent to that of rHla. Aliquots of rHla and rH35L were stored in the vapor phase of liquid nitrogen.

### 4.3. Human Airway Model Epithelial Cell Cultures and Culture Conditions

Experiments were performed with two immortalized human airway epithelial cell lines (16HBE14o^-^, S9) and one alveolar cancer cell line (A549). With permission of D.C. Gruenert 16HBE14o^-^ cells were received from K. Kunzelmann (University of Regensburg, Germany). S9 cells were purchased from ATCC-LGC Standards (Wesel, Germany, S9). A549 cells were obtained from the cell collection of the Friedrich Loeffler-Institute (Riems, Germany).

Cells were cultured on 10 cm Cell+ dishes (3902300, Sarstedt, Numbrecht, Germany) in Eagle’s minimal essential medium (MEM) (P03-2950, PAN-Biotech, Aidenbach, Germany) containing 10% FBS superior (S0615, Merck, Darmstadt, Germany), 29.8 mmol/L NaHCO_3_ and 1% (*w*/*v*) penicillin/streptomycin solution (P06-07050, PAN-Biotech, Aidenbach, Germany) at 37 °C and gassed with 5% CO_2_. Cell culture medium of A549 cells additionally contained 1% L-glutamine solution (P04-80100, PAN-Biotech, Aidenbach, Germany). Cell culture medium was changed every 3 days. Just before cells formed confluent monolayers, they were passaged onto new cell culture dishes or used directly for experiments. All cell cultures were checked for *Mycoplasma* contaminations on a regular basis.

### 4.4. Abundance of rHla-Heptamers in 16HBE14o^-^, S9 or A549 Cells upon Pulse-Treatment with Monomeric rHla

S9 cells were treated with 2000 ng/mL and 16HBE14o^-^ and A549 cells with 500 ng/mL rHla or with PBS as negative control for 90 min. These previously examined rHla concentrations still showed a toxic effect after 90 min of treatment, but these cells were able to recover, so that they should be able to process the toxin. Afterwards, cells were washed with PBS and incubated in fresh cell culture medium for 0, 1, 2, 4, 6 or 8 h. After washing cells again with PBS, plasma membrane preparations, to investigate a removal of toxin heptamers from the plasma membrane, or whole cell lysates, to check for a complete elimination of Hla-heptamers in cells, were generated. The preparation of whole cell extracts was performed as described previously [12]. For generating plasma membrane preparations 1 mL of hypotonic solution (10 mmol/L triethanolamine, 4.5 mmol/L EDTA tetrasodium salt dihydrate, pH 6.8, containing aprotinin (0.31 mmol/L), leupeptin (4.21 μmol/L) and pep-statin (2.92 μmol/L), as well as 1 mmol/L PMSF and 0.33 mmol/L ortho-vanadate) was added to cells, which were then subsequently scraped off the cell culture dish and transferred to a 1.5 mL reaction vessel. Cells were lysed by rapidly freezing in liquid nitrogen and thawing in a water bath three 3 times. Lysates were centrifuged at 16,200× *g* for 3 min at 4 °C. Supernatants were discarded and pellets were resuspended in 200 µL hypotonic solution. A small portion of the samples were used for determination of protein concentration [46]. The remaining volume of samples were mixed with the same volume of SDS/Chol sample buffer (49.5 mmol/L TRIS, pH adjusted to 6.8, 40% Glycerol [*w*/*v*], 4% β-mercapto-ethanol [*w*/*v*], 1% SDS [*w*/*v*], 500 µmol/L sodium deoxycholate, 580 µmol/L bromophenol blue sodium salt), aliquoted and frozen at −80 °C until use.

For the detection of the rHla heptamers semi-dry blotting (previously described [23]) or wet blotting (previously described [12]) were used. Since stained protein bands in membrane preparation samples were used to normalize the results, nitrocellulose membranes stained with Ponceau S were carefully washed with A. dest and stained protein bands were scanned with the x-finity pro 42 scanner (Quatographic, Braunschweig, Germany). 

Blots were blocked with 3% nonfat dried milk. For detection of rHla heptamers, blots were incubated with a Hla primary antibody (1:3,333) at 4 °C overnight and with a goat anti-rabbit IgG-HRP secondary antibody (1: 5,882) for 1 h at room temperature. Signals were detected by using a chemiluminescence substrate (AdvanstaWesternBright, Biozym, Hess. Oldendorf, Germany) and a Fusion FX7-SL gel imager (VilbertLourmat, Eberhardzell, Germany) or IntasChemostar ECL imager (Intas, Göttingen, Germany).

Phoretix 1 D was utilized to measure the density of protein bands (Nonlinear Dynamics, Newcastle upon Tyne, UK). To correct for possible minor differences due to different exposure times, the average density of all bands on each membrane image was used to normalize the densities of the individual bands of the same membrane. Signal intensities of rHla heptamere bands of membrane preparations samples were normalized to intensities of stained protein bands and whole cell lysate samples of bands of the housekeeping protein β-actin.

### 4.5. Continuous Iodixanol Density Gradients

Five nearly confluently grown 10 cm cell culture dishes from each of the 3 cell lines per sample were required for the experiment. All three cell lines were treated with 2000 ng/mL rHla for 2.5 h or as a control with the mutant rH35L. After treatment cells were washed twice with ice cold 5 mL PBS and 200 µL of the homogenization buffer (250 mmol/L D(+)-sucrose, 50 mmol/L HEPES, 78 mmol/L KCl, 4 mmol/L MgCl_2_·6H_2_O, 8.4 mmol/L CaCl_2_·2H_2_O, 10 mmol/L EGTA, pH 7.0 adjusted with 1 mol/L KOH, containing aprotinin (0.31 mmol/L), leupeptin (4.21 μmol/L) and pep-statin (2.92 μmol/L), as well as 1 mmol/L PMSF and 0.33 mmol/L ortho-vanadate) was pipetted onto the cells. Cells were then scraped from the dish and merged in a 2 mL reaction vessel and broken up by using a 27 G syringe needle. Post nuclear supernatant (PNS) of cell homogenates were generated as described [27].

A gradient former (BioRad, Munich, Germany) was used to produce 10 mL of a continuous OptiPrep™ density gradient between 5% and 20% (5%/20% OptiPrep™, 50 mmol/L HEPES, 78 mmol/L KCl, 4 mmol/L MgCl_2_·6H_2_O, 8.4 mmol/L CaCl_2_·2H_2_O, 10 mmol/L EGTA, pH 7.0 adjusted with 1 mol/L KOH, containing aprotinin (0.31 mmol/L), leupeptin (4.21 μmol/L) and pep-statin (2.92 μmol/L), as well as 1 mmol/L PMSF and 0.33 mmol/L ortho-vanadate) in a 12 mL Seton Open-Top UltraCote Centrifuge Tube [14 × 89 mm] (Science Services, Munich, Germany). The respective sample (1 mL) was carefully layered on the gradient and were centrifuged in a SW 40 Ti rotor (Beckman Coulter, Krefeld, Germany) at a speed of 90,000× *g* and 4 °C in a Optima™ L-80 ultracentrifuge (Beckman Coulter, Krefeld, Germany) for 20 h [27]. Fractions of 500 µL were taken from the ultracentrifuge tubes from top to bottom and mixed with the same volume of SDS sample buffer, aliquoted and frozen at −80 °C until further use.

Fractions (25 µL) were separated by SDS/PAGE (10% or 13% gel) in a PROTEAN II xi cell system (BioRad, Munich, Germany) with water cooling and on ice, and proteins were transferred onto nitrocellulose membrane (HP40, Roth, Karlsruhe, Germany) by semi-dry blotting [23] for 1 h. Primary antibodies against Hla (1:3333) for the detection of rHla monomers and heptamers, against the early endosome marker protein EEA1 (1:500), against the late endosome and autophagosome marker protein Rab7 (1:6666), and as a control for contamination of fractions with plasma membrane fragments against the alpha subunit (SU) of Na^+^K^+^-ATPase (1:1000) were used for quantification. Depending on the primary antibodies, a mouse IgGκ light chain binding protein -HRP (1:3333), a horse anti-mouse IgG-HRP (1:6666) or a goat anti-rabbit IgG-HRP (1:6666) were used as secondary antibodies.

### 4.6. Fluorescence Microscopy

16HBE14o^-^, S9 and A549 cells were cultivated on precision coverslips (Ø 18 mm) in a 12-well plate up to a coverage of approximately 70%. Cells were treated for 2 h with 2000 ng/mL rHla or as control with PBS and then washed twice with 1 mL of a washing solution (1.3 mmol/L CaCl_2_·2H_2_O, 1.5 mmol/L MgCl_2_·6H_2_O, dissolved in PBS, auto-claved and filtered, pH 7.4). Cells were fixed with 1 mL 4% formaldehyde at 4 °C for 20 min, washed once with 1 mL filtered PBS and were permeabilized with 1 mL 0.05% saponin in filtered PBS twice for 15 min at room temperature. After washing cells twice for 5 min with 1 mL filtered PBS, nonspecific antibody binding sites were blocked with 1 mL of blocking solution (5% BSA in filtered PBS) for 1 h at 4 °C. Cells were incubated with the StrepMAB-Classic (anti-Strep-tag II antibody) Chromeo 546 conjugate antibody (1:50 in blocking solution), which could bind to the not cleaved Strep-tag II of rHla, overnight at 4 °C in a humid chamber and then washed five times for 5 min with 1 mL filtered PBS. Cell nuclei were stained with DAPI and then cells were mounted with Mowiol 4-88 with 1,4-Diazabicyclo[2.2.2]octan (DABCO) onto microscope slides.

A LEICA DMi8 microscope with a 63× oil immersion objective (Leica, Wetzlar, Germany) and LAS X software (version 3.4.2.18368) was used to acquire immunofluorescence images. For the detection of the Strep-tag II of rHla a Texas Red filter set and a DAPI filter set for the detection of cell nuclei was used. The settings for the detection of rHla, such as exposure time and brightness, were always the same for all three cell lines.

### 4.7. Potential Proteasomal or Lysosomal rHla Heptamer Degradation in S9 Cells

S9 cells were incubated for 1 h with 100 nmol/L bafilomycin A1 or 100 µmol/L chloroquine as lysosomal inhibitors or with 10 µmol/L Mg-132 or 10 nmol/L bortezomib as proteasomal inhibitors or as controls with DMSO (solvent for bafilomycin, Mg-132 and bortezomib) in cell culture medium. Cells were then treated with 2000 ng/mL rHla for 90 min or as negative control with PBS and washed twice with 5 mL PBS. Fresh cell culture medium containing the respective inhibitor or DMSO was added to the cells. After 0 or 8 h incubation whole cell lysates were prepared and wet blotting [12] were used to verify the removal of rHla heptamers as described above. The used concentrations of these inhibitors were previously tested for their effects.

### 4.8. Stability of rHla Heptamers at an Acidic pH

16HBE14o^-^ cells were treated with 2000 ng/mL rHla for 1 h and then washed twice with ice-cold PBS. Whole cell lysates were prepared with a pH neutral lysis buffer (see standard lysis buffer for the preparation of whole cell lysates) or an acidic lysis buffer (100 mmol/L KCl, 20 mmol/L NaCl, 2 mmol/L MgCl_2_, 0.96 mmol/L NaH_2_PO_4_, 0.84 mmol/L CaCl_2_, 1 mmol/L EGTA, 22.7 mmol/L sodium acetate trihydrate, 77.3 mmol/L acetic acid, 0.5% Tween 20 (*v*/*v*), pH 4, containing aprotinin (0.31 mmol/L), leupeptin (4.21 μmol/L) and pep-statin (2.92 μmol/L), as well as 1 mmol/L PMSF and 0.33 mmol/L ortho-vanadate). Lysates were used to determine the amount of rHla heptamers via semi-dry blotting. Since some proteins were precipitated at acidic pH, results were not standardized to β-actin but to the sample volume applied to the respective lane.

### 4.9. Mass Spectrometric Analysis of Proteins in Pelleted Extracellular Vesicles in rHla-Treated or in Serum-Starved S9 Cells

S9 cells were cultivated on 10 cm cell culture dishes up to a confluence of >90% in a serum-free growth medium for respiratory epithelial cells (C-21060, PromoCell, Heidelberg, Germany). This was to prevent exosomes from the otherwise used fetal calf serum from being detected in the cell culture medium and causing false positive results. These S9 cells were treated with 2000 ng/mL rHla for 90 min and then washed twice with 10 mL PBS to remove unbound rHla completely. Fresh growth medium (10 mL) was added to the cells and they were cultivated for 8 h. S9 cells treated with PBS for 90 min and then incubated for 8 h and 48 h in a starvation medium (base medium without fetal calf serum) served as controls. To extract possible extracellular vesicles from cell culture media, differential ultracentrifugation was used, as described previously [47]. Pellets were dissolved in 200 µL 5% SDS in A. bidet. Samples were transferred to 1.6 mL low binding reaction vessels and frozen at −80 °C until further use.

To extract the proteins for MS analysis, samples were shaken twice for 10 min with 3 min ultrasonication in between. Afterwards, centrifugation at 17,000× *g* for 10 min at room temperature was performed and supernatants were transferred to a new 1.5 mL low binding reaction vessel and MgCl_2_ stock solution was added to a final concentration of 4 mmol/L to the samples. To cleave interfering DNA samples were treated with 0.005 U/µL benzonase and subsequently incubated in an ultrasonic bath for 5 min and then centrifuged for 30 min at 17,000× *g*. Supernatants were then transferred to new 1.5 mL low binding reaction vessels and the protein concentrations were determined by using a Micro BCA Assay (Pierce Micro BCATM Protein Assay Kit, Sigma Aldrich, St. Louis, MO, USA) and a NanoDrop™ 8000 spectrophotometer (Thermo Scientific™, Waltham, MA, USA) according to manufacturer’s instruction. The SP3 protocol (SP3 = single pot solid-phase enhanced sample preparation) was used for on-bead protein digestion and peptide purification [48]. In brief, 4 μg protein per sample were bound to a mix of carboxylate modified hydrophilic and hydrophobic magnetic beads on which the proteins were cleaved with trypsin (protein: protease ratio 25:1). Peptides were then eluted with 2% DMSO and measured with a Q Exactive™ Plus mass spectrometer (Thermo Fisher Scientific, Bonn, Germany) in data independent acquisition (DIA) mode as previously described [49].

To identify the proteins from the MS data, the software Spectronaut™ 13 (v13.13.200417.43655) (Biognosys AG, Schlieren, Switzerland) was used in directDIA™ mode against the Uniprot protein database limited to human protein entries (04/2019) supplemented with the sequence of *S. aureus* rHla. Therefore, trypsin/P was set as digestion enzyme with two missed cleavages allowed and oxidation of methionine was enabled as variable modification. Global sparse imputation based on protein q-value cutoff of 0.001 was set as analysis strategy. Statistical analysis was performed using R version 4.0.2 (2020-06-22). Raw data were median normalized over the MS2 total peak area intensities (EG.TotalQuantity) for ions with a q-value cutoff <0.001. Missing values were replaced using the half-minimal intensity value from the whole dataset and methionine oxidized peptides were excluded.

### 4.10. Data Presentation and Statistics

Data are presented as means ± S.D. of n experiments on different cell preparations. Significant differences in the series of means were detected by ANOVA. Means were tested for significant differences to the appropriate controls using Student’s *t*-test, if variances were equal, or otherwise Welch’s *t*-test was used. Significant differences of means were presented as: *p* < 0.05 (*), *p* < 0.01 (**) and *p* < 0.001 (***).

## Figures and Tables

**Table 1:** Exosome marker proteins detected by mass spectrometry in supernatants of rHla-treated or serum-starved S9 cells. Extracellular vesicles were purified from cell culture media of S9 cells cultured in a serum-free growth medium and treated with 2000 ng/mL rHla (treated with rHla) or from culture medium of S9 cells that had been serum-starved for 8 h (8 h in starvation medium) or 48 h (48 h ion starvation medium). Vesicle fractions were prepared by differential centrifugation, pelleted by ultracentrifugation, and analyzed by electrospray ionization (ESI) mass spectrometry. Protein sequences were checked against the Uniprot protein database (04/2019), also including the *S. aureus* rHla sequence. Marker proteins for exosomes [36,37] that were detected in at least one of the three analyzed samples were listed together with the respective numbers of identified peptides of these proteins.

Category	SwissProt #	Protein Description	Treated with rHla	8 h in Starvation Medium	48 h in Starvation Medium
ESCRT	Q99816	Tumor susceptibility gene 101 protein, TSG101	8	7	10
	Q9H9H4	Vacuolar protein sorting-associated protein 37B, VPS37B	4	4	4
	A5D8V6	Vacuolar protein sorting-associated protein 37C, VPS37C	0	0	2
	O43633	Charged multivesicular body protein 2a, CHMP2A	4	4	5
ESCRT-associated	Q8WUM4	Programmed cell death 6 interacting protein, PDCD6IP/Alix	34	31	35
Tetraspanins	P08962	CD63 antigen, CD63	0	3	3
	P21926	CD9 antigen, CD9	5	6	6
	P27701	CD82 antigen, CD82	2	4	4
	P48509	CD151 antigen, CD151	2	3	4
	O43657	Tetraspanin-6, TSPNA6	0	0	2
RNA binding protein, ribosomal protein	P26373	60S ribosomal protein L13, RPL13	9	10	10
	P61254	60S ribosomal protein L26, RPL26	0	0	2
	P62851	40S ribosomal protein S25, RPS25	6	6	7
Cargo selection	P46934	E3 ubiquitin-protein ligase NEDD4, NEDD4	0	0	2
	Q96PU5	E3 ubiquitin-protein ligase NEDD4-like, NEDD4L	5	5	7
Protein trafficking, protein sorting	O00560	Syntenin-1, Syndecan-binding protein 1, SDCBP	10	10	10
	Q8N5I2	Arrestin domain-containing protein 1, ARRDC1	2	3	4
	Q9H0U4	Ras-related protein Rab-1B, Rab1B	12	11	11
	P20339	Ras-related protein Rab-5A, Rab5A	8	8	9
	P61020	Ras-related protein Rab-5B, Rab5B	6	6	6
	P61006	Ras-related protein Rab-8A, Rab8A	6	6	6
	Q15907	Ras-related protein Rab-11B, Rab11B	12	10	12
Integral membrane protein	P06756	Integrin alpha-V, ITGAV	12	14	16
	O14672	A disintegrin and metalloproteinase domain-containing protein 10, ADAM10	7	5	7
	Q969P0	Immunoglobulin superfamily member 8, IGSF8	10	10	10
GPI-anchor	P35052	Glypican-1, GPC1	9	8	9
Scaffolding protein	O75955	Flotillin-1, FLOT1	19	17	20
	P11142	Heat shock 70 kDa protein 8, HSPA8	33	32	35

**Table 2:** Micro-vesicle marker proteins detected by mass spectrometry in supernatants of rHla-treated or serum-starved S9 cells. S9 cells were treated and micro-vesicles were prepared and analyzed as described in the legend to Table 1. Marker proteins for micro-vesicles [36,37] that were detected in at least one of the three analyzed samples were listed together with the respective numbers of identified peptides of these proteins.

Category	Swiss Prot #	Protein Description	Treated with rHla	8 h in Starvation Medium	48 h in Starvation Medium
Ribonuclear protein	P31943	Heterogeneous nuclear ribonucleoprotein H, HNRNPH1	10	7	10
	P14866	Heterogeneous nuclear ribonucleoprotein L, HNRNPl	14	11	15
Calcium-bindingchaperone	P27797	Calreticulin, CALR	8	9	9
Nuclear export receptor	P55060	Exportin-2, CSE1L	28	21	29
Calcium-dependent phospholipid binding protein	O75131	Copine-3, CPNE3 protein, CPNE3	7	6	7
Mitochondrial outer membrane protein	P21796	Voltage-dependent anion-selective channel protein 1, VDAC1	7	4	8
	P45880	Voltage-dependent anion-selective channel protein 2, VDAC2	6	6	6
Mitochondrial andnuclear protein	Q99623	Prohibitin-2, PHB2	6	6	8
Mitochondrial inner membrane protein	P48047	ATP synthase subunit O, mitochondrial, ATP5O	0	0	5
	Q00325	Solute carrier family 25 member 3, SLC25A3	3	4	4
	P25705	ATP synthase subunit alpha, mitochondrial, ATP5A1	26	16	24
ABC transporter	P61221	ATP-binding cassette sub-family E member 1, ABCE1	11	6	11
Integral membraneprotein	Q07065	Cytoskeleton-associated protein 4, CKAP4	6	12	16
Cytoskeleton/microtubule regulation, cell motility	Q9UM54	Unconventional myosin-VI, MYO6	8	6	12
	O75369	Filamin-B, FLNB	61	65	80
	Q9H0H5	Rac GTPase-activating protein 1, RACGAP1	8	9	12
	Q02241	Kinesin-like protein, KIF23	12	16	19
	P06753	Tropomyosin alpha-3 chain, TPM3	6	5	6
Cytosolic enzymes	P49588	Alanine-tRNA ligase, cytoplasmic, AARS	24	18	25
	P31939	Bifunctional purine biosynthesis protein, ATIC	21	17	25
Enzyme, organizer of 3D structure of proteins	Q96HE7	ERO1-like protein alpha, ERO1L	5	5	7
	P13667	Protein disulfide-isomerase A4, PDIA4	11	15	17
Beta-subunit of heterotrimeric protein	P62879	Guanine nucleotide-binding protein G(I)/G(S)/G(T) subunit beta-2, GNB2	7	5	7

## Data Availability

Original data are deposited at BioStudies (EMBL-EBI) (https://www.ebi.ac.uk/biostudies/ (accessed on 19 February 2021)) under the accession number S-BSST585. The mass spectrometry proteomics data that have been deposited to the ProteomeXchange Consortium via the PRIDE [50] partner repository with the dataset identifier PXD023678.

## Supplementary Materials

The following are available online at https://www.mdpi.com/2072-6651/13/3/173/s1, Table S1, Table S2: Comparisons of exosome marker proteins detected by mass spectrometry in supernatants of rHla-treated or serum-starved S9 cells, Table S3: Comparisons of microvesicle marker proteins detected by mass spectrometry in supernatants of rHla-treated or serum-starved S9 cells.
