# Supplementary material for: Major Determinants of Airway Epithelial Cell Sensitivity to S. aureus Alpha-Toxin: Disposal of Toxin Heptamers by Extracellular Vesicle Formation and Lysosomal Degradation
Source: Toxins (Basel). 2021 Feb 24;13(3):173. doi: 10.3390/toxins13030173 (PMC7996177; doi:10.3390/toxins13030173)
Supplement: Supplementary file 1 [file toxins-13-00173-s001.zip › toxins-1111098_Tab S2_S3_final.pdf]

Major Determinants of Airway Epithelial Cell Sensitivity to *S. aureus* Alpha-Toxin: Disposal of Toxin Heptamers by Extracellular Vesicle Formation and Lysosomal Degradation

Nils Möller, Sabine Ziesemer, Christian Hentschker, Uwe Völker and Jan-Peter Hildebrandt

Table S2. Comparisons of exosome marker proteins detected by mass spectrometry in supernatants of rHla-treated or serum-starved S9 cells. Extracellular vesicles were purified from cell culture media of S9 cells cultured in a serum-free growth medium and treated with 2000 ng/ml rHla (treated with rHla) or from culture medium of S9 cells that had been serum-starved for 8 h (8 h in starvation medium) or 48 h (48 h ion starvation medium). Vesicle fractions were prepared by differential centrifugation, pelleted by ultracentrifugation, and analyzed by electrospray ionization (ESI) mass spectrometry. Protein sequences were checked against the Uniprot protein database (04/2019) also including the S. aureus rHla sequence. Median normalized Hi3 peptide intensities of the detected proteins were compared between the different treatments and the expression differences were presented as signal log2 ratio. Shown are proteins, which were identified as marker proteins for exosomes [1,2] and found in all three treatments ($n = 3$). The signal log2-ratios are presented as: ≥ 4 = + + (≥ 16-fold increased), ≥ 2 = + + (≥ 4-fold increased), ≥ 1 = + (≥ 2-fold increased), 0 (same or little increased or decreased expression), ≤ -1 = - (≤ 1/2 of the comparison value), ≤ -2 = - - (≤ 1/4 of the comparison value), ≤ -4 = - - - (≤ 1/16 of the comparison value) (The respective values of the signal log2-ratios and the significant differences are shown in Table S3).

Category	SwissProt #	Protein Description	Signal log2 Ratio		
			Treated with rHla / 8 h in Starvation Medium	Treated with rHla / 48 h in Starvation Medium	8 h in Starvation Medium / 48 h in Starvation Medium
endosomal sorting complexes required for transport (ESCRT)	Q99816	Tumor susceptibility gene 101 protein, TSG101	+	-	- -
	Q9H9H4	Vacuolar protein sorting-associated protein 37B, VPS37B	+	-	- -
	O43633	Charged multivesicular body protein 2a, CHMP2A	0	- -	- -
ESCRT-associated	Q8WUM4	Programmed cell death 6 interacting protein, PDCD6IP/Alix	+	0	- -
Tetraspanin	P21926	CD9 antigen, CD9	-	- - -	- -
	P27701	CD82 antigen, CD82	0	- -	- -
	P48509	CD151 antigen, CD151	0	- -	- -
RNA binding protein, ribosomal protein	P26373	60S ribosomal protein L13, RPL13	0	- -	- -
	P62851	40S ribosomal protein S25, RPS25	0	- -	- -
cargo selection	Q96PU5	E3 ubiquitin-protein ligase NEDD4-like, NEDD4L	+	-	- -
	O00560	Syntenin-1, Syndecan-binding protein 1, SDCBP	+	- -	- -

protein trafficking, protein sorting	Q8N5I2	Arrestin domain-containing protein 1, ARRDC1	0	- -	- -
	Q9H0U4	Ras-related protein Rab-1B, Rab1B	+ + +	+	- -
	P20339	Ras-related protein Rab-5A, Rab5A	+ +	+	-
	P61020	Ras-related protein Rab-5B, Rab5B	+ + +	+ +	- -
	P61006	Ras-related protein Rab-8A, Rab8A	+ +	0	- -
	Q15907	Ras-related protein Rab-11B, Rab11B	+ +	0	- -
integral membrane protein	P06756	Integrin alpha-V, ITGAV	0	- -	- -
	O14672	A disintegrin and metalloproteinase domain-containing protein 10, ADAM10	+	- -	- -
	Q969P0	Immunoglobulin superfamily member 8, IGSF8	+	-	- -
GPI-anchor	P35052	Glypican-1, GPC1	+	0	- -
scaffolding protein	O75955	Flotillin-1, FLOT1	+	-	- -
	P11142	Heat shock 70 kDa protein 8, HSPA8	+ +	-	- - -

1. Xu, R.; Greening, D.W.; Zhu, H.J.; Takahashi, N.; Simpson, R.J. Extracellular vesicle isolation and characterization: toward clinical application. *J. Clin. Invest.* **2016**, *126*, 1152-1162. doi:10.1172/JCI81129.

2. Théry, C.; Witwer, K.W.; Aikawa, E.; Alcaraz, M.J.; Anderson, J.D.; Andriantsitohaina, R.; Antoniou, A.; Arab, T.; Archer, F.; Atkin-Smith, G.K.; et al. Minimal information for studies of extracellular vesicles 2018 (MISEV2018): a position statement of the International Society for Extracellular Vesicles and update of the MISEV2014 guidelines. *J. Extracell Vesicles* **2018**, *7*, 1535750. doi:10.1080/20013078.2018.1535750.

Table S3. Comparisons of micro-vesicle marker proteins detected by mass spectrometry in supernatants of rHla-treated or serum-starved S9 cells. Extracellular vesicles were purified from cell culture media of S9 cells cultured in a serum-free growth medium and treated with 2000 ng/ml rHla (treated with rHla) or from culture medium of S9 cells that had been serum-starved for 8 h (8 h in starvation medium) or 48 h (48 h ion starvation medium). Vesicle fractions were prepared by differential centrifugation, pelleted by ultracentrifugation, and analyzed by electrospray ionization (ESI) mass spectrometry. Protein sequences were checked against the Uniprot protein database (04/2019) also including the S. aureus rHla sequence. Median normalized Hi3 peptide intensities of the detected proteins were compared between the different treatments and the expression differences were presented as signal log2 ratio. Shown are proteins, which were identified as marker proteins for micro-vesicles [1,2] and found in all three treatments (*n* = 3). The signal log2-ratios are presented as: ≥ 4 = + + (≥ 16-fold increased), ≥ 2 = + + (≥ 4-fold increased), ≥ 1 = + (≥ 2-fold increased), 0 (same or little increased or decreased expression), ≤ -1 = - (≤ 1/2 of the comparison value), ≤ -2 = - - (≤ 1/4 of the comparison value), ≤ -4 = - - - (≤ 1/16 of the comparison value) (The respective values of the signal log2-ratios and the significant differences are shown in Table S3).

**Category**	**SwissProt #**	**Protein Description**	**Signal log2 Ratio**		
			**Treated with rHla / 8 h in Starvation Medium**	**Treated with rHla / 48 h in Starvation Medium**	**8 h in Starvation Medium / 48 h in Starvation Medium**
ribonuclear protein	**P31943**	Heterogeneous nuclear ribonucleoprotein H, HNRNPH1	+ +	0	- -
	P14866	Heterogeneous nuclear ribonucleoprotein L, HNRNPl	+ +	-	- -
calcium-binding chaperone	P27797	Calreticulin, CALR	+	- -	- -
nuclear export receptor	P55060	Exportin-2, CSE1L	+ +	-	- - -
calcium-dependent phospholipid binding protein	O75131	Copine-3, CPNE3 protein, CPNE3	0	- -	- -
mitochondrial outer membrane protein	P21796	Voltage-dependent anion-selective channel protein 1, VDAC1	+	0	- -
	P45880	Voltage-dependent anion-selective channel protein 2, VDAC2	+ +	0	- -

mitochondrial and nuclear protein	Q99623	Prohibitin-2, PHB2	0	- -	- -
mitochondrial inner membrane protein	Q00325	Solute carrier family 25 member 3, SLC25A3	+ +	0	- -
	P25705	ATP synthase subunit alpha, mitochondrial, ATP5A1	+ + +	+ +	- -
ABC transporter	P61221	ATP-binding cassette sub-family E member 1, ABCE1	+ + +	0	- - -
integral membrane protein	Q07065	Cytoskeleton-associated protein 4, CKAP4	+	-	- -
cytoskeleton/microtubule regulation, cell motility	Q9UM54	Unconventional myosin-VI, MYO6	+ +	0	- - -
	O75369	Filamin-B, FLNB	+ +	-	- -
	Q9H0H5	Rac GTPase-activating protein 1, RACGAP1	+	- -	- -
	Q02241	Kinesin-like protein, KIF23	0	- -	- -
	P06753	Tropomyosin alpha-3 chain, TPM3	+ +	0	- -
cytosolic enzyme	P49588	Alanine--tRNA ligase, cytoplasmic, AARS	+ +	0	- -
	P31939	Bifunctional purine biosynthesis protein, ATIC	+ + +	+	- - -
enzyme, organizer of 3D structure of proteins	Q96HE7	ERO1-like protein alpha, ERO1L	+ +	-	- -
	P13667	Protein disulfide-isomerase A4, PDIA4	0	- -	- -
membrane-associated protein, beta-subunit of heterotrimeric protein	P62879	Guanine nucleotide-binding protein G(I)/G(S)/G(T) subunit beta-2, GNB2	+ +	0	- -

1. Xu, R.; Greening, D.W.; Zhu, H.J.; Takahashi, N.; Simpson, R.J. Extracellular vesicle isolation and characterization: toward clinical application. *J. Clin. Invest.* **2016**, *126*, 1152-1162. doi:10.1172/JCI81129.

2. Théry, C.; Witwer, K.W.; Aikawa, E.; Alcaraz, M.J.; Anderson, J.D.; Andriantsitohaina, R.; Antoniou, A.; Arab, T.; Archer, F.; Atkin-Smith, G.K.; et al. Minimal information for studies of extracellular vesicles 2018 (MISEV2018): a position statement of the International Society for Extracellular Vesicles and update of the MISEV2014 guidelines. *J. Extracell Vesicles* **2018**, *7*, 1535750. doi:10.1080/20013078.2018.1535750.
